# Multi-modal artificial intelligence for the combination of automated 3D breast ultrasound and mammograms in a population of women with predominantly dense breasts

**DOI:** 10.1186/s13244-022-01352-y

**Published:** 2023-01-16

**Authors:** Tao Tan, Alejandro Rodriguez-Ruiz, Tianyu Zhang, Lin Xu, Regina G. H. Beets-Tan, Yingzhao Shen, Nico Karssemeijer, Jun Xu, Ritse M. Mann, Lingyun Bao

**Affiliations:** 1 Department of Radiology, Netherlands Cancer Institute (NKI), Plesmanlaan 121, 1066 CX Amsterdam, The Netherlands; 2 Faculty of Applied Science, Macao Polytechnic University, Macao, 999078 China; 3 ScreenPoint Medical, 6525 EC Nijmegen, The Netherlands; 4 GROW School for Oncology and Development Biology, Maastricht University, P. O. Box 616, 6200 MD Maastricht, The Netherlands; 5 School of Information Science and Technology, ShanghaiTech University, Shanghai, 201210 China; 6 Affiliated Hangzhou First People’s Hospital, Zhejiang University School of Medicine, Xueshi Road, Hubin Street, Shangcheng District, Hangzhou, 310006 Zhejiang China; 7 Department of Diagnostic Imaging, Radboud University Medical Center, PO Box 9101, 6500 HB Nijmegen, The Netherlands; 8 Institute for AI in Medicine, School of Artificial Intelligence, Nanjing University of Information Science and Technology, Nanjing, 210044 China

**Keywords:** Mammography, Automated 3D breast ultrasound, Artificial intelligence, Breast cancer, Deep learning

## Abstract

**Objectives:**

To assess the stand-alone and combined performance of artificial intelligence (AI) detection systems for digital mammography (DM) and automated 3D breast ultrasound (ABUS) in detecting breast cancer in women with dense breasts.

**Methods:**

430 paired cases of DM and ABUS examinations from a Asian population with dense breasts were retrospectively collected. All cases were analyzed by two AI systems, one for DM exams and one for ABUS exams. A selected subset (*n* = 152) was read by four radiologists. The performance of AI systems was based on analysis of the area under the receiver operating characteristic curve (AUC). The maximum Youden’s index and its associated sensitivity and specificity were also reported for each AI systems. Detection performance of human readers in the subcohort of the reader study was measured in terms of sensitivity and specificity.

**Results:**

The performance of the AI systems in a multi-modal setting was significantly better when the weights of AI-DM and AI-ABUS were 0.25 and 0.75, respectively, than each system individually in a single-modal setting (AUC-AI-Multimodal = 0.865; AUC-AI-DM = 0.832, *p* = 0.026; AUC-AI-ABUS = 0.841, *p* = 0.041). The maximum Youden’s index for AI-Multimodal was 0.707 (sensitivity = 79.4%, specificity = 91.2%). In the subcohort that underwent human reading, the panel of four readers achieved a sensitivity of 93.2% and specificity of 32.7%. AI-multimodal achieves superior or equal sensitivity as single human readers at the same specificity operating points on the ROC curve.

**Conclusion:**

Multimodal (ABUS + DM) AI systems for detecting breast cancer in women with dense breasts are a potential solution for breast screening in radiologist-scarce regions.

## Key points

1. Combining AI systems from different modalities may increase the overall performance.
2. Combined AI outperforms readers from single modalities for breast cancer detection.
3. Combined AI can be an alternative to radiologists in breast imaging reading.

## Background

Breast cancer is the most frequent cancer in women worldwide. The prognosis of breast cancer strongly depends on the stage of the disease [1]. Population-based screening programs have been implemented in many countries to detect breast cancer at an early stage. Generally, in these programs, women above a certain age are invited periodically to undergo a breast imaging exam. The primary modality of examination for screening is usually digital mammography (DM), a 2D imaging technique [2]. However, DM has limitations to detect breast cancer in women with dense breasts because of tissue superposition [3]. Multi-modal machine learning or deep learning has been a hot research topic in recent years and it is a currently trend as multi-modalities data could potentially provide more useful complementary information for cancer diagnosis [4–6].

These limitations can be overcome by adding supplemental imaging during screening. In Japan, evidence from a screening randomized controlled trial [7], suggested that adjunctive breast ultrasound examination (US) increases sensitivity and detection rate of early cancers. Automated 3D breast ultrasound (ABUS) has the advantage of automated operation allowing standardized image acquisition by technologists and has been proposed as a supplementary screening modality to DM in populations of women with predominantly dense breasts [8]. It was already shown that ultrasound can yield additional cancer detection of about 4.2/1000 [9]. ABUS is potentially suitable for screening, and may be of particular interest in Asian countries or the Asian population in western countries where most women have dense breasts. Currently, the application of ABUS for screening in Asia is under investigation [10].

Due to the large numbers of women that are screened, current screening programs with DM are already very labor-intensive. And even when double reading is performed, up to 25% of cancers are not detected [11]. The need to improve the quality and cost-efficiency of screening has triggered the development of computer-aided detection (CAD) systems. These have been available for DM for many years, and have more recently also become available for ABUS.

CAD systems have improved in detection performance by using deep-learning-based artificial intelligence (AI) algorithms. In breast imaging some of these CAD systems perform at the level of experienced breast radiologists [12]. Concurrent use of AI systems offers radiologists the possibility to know the output of AI while reading a case, an approach that has been demonstrated to improve readers performance in both ABUS [13–15] and DM [16]. With the performance of AI system approaching that of radiologists [17, 18], it may be feasible to use AI tools for automated and safe triaging of women in screening with DM [19, 20]. However, so far, no studies have been performed that explore the potential of combining AI systems for different modalities, such as DM and ABUS in order to improve the detection of breast cancer and/or triage populations in a multi-modal breast cancer screening program.

The objective of this study was to evaluate the potential of combining the automated detections of two commercially available AI systems approved by the U.S. Food and Drug Administration (FDA), one for DM [17] and one for ABUS [8] in terms of breast cancer detection performance as well as benchmarking their stand-alone performance compared to radiologists’ in a population of women with predominantly dense breasts.

## Methods

### Case collection

Paired studies from women undergoing both bilateral digital mammography (DM) and automated 3D breast ultrasound (ABUS) examination were consecutively collected from a single institution in China between 2016 and 2018. Women attended this hospital for breast imaging after developing symptoms or, without symptoms, for a self-motivated screening breast examination. Cases were excluded from the final cohort when: the main suspicious finding in DM was calcifications, women were pregnant, breastfeeding or planning to become pregnant, women had a prior diagnosis of/or were treated for breast cancer, women had breast implants or another history of breast augmentation. This resulted in an eligible cohort of 430 cases.

The use of patient electronic health records in this study was approved by Affiliated Hangzhou First People's Hospital, Zhejiang University School of Medicine. Written informed consent was obtained from all patients. Patient and lesion characteristics are listed in Table 1. In total 42 malignant, 114 benign, and 274 normal cases were collected. Breast density was classified as heterogeneously or extremely dense in 73% of patients (BI-RADS categories c and d). All malignant and benign cases were confirmed by biopsy. Normal cases were confirmed by at least one year of negative follow-up with ABUS and DM.

**Table 1:** Characteristics of the cases collected for the study

	Full cohort (*n* = 430)	Observer study Cohort (*n* = 152)
Age (y)	48 (30–70)	50 (33–70)
DM compressed breast thickness (mm)	47 (19–78)	48 (20–76)
BI-RADS density category	A: 10 B: 106 C: 217 D: 97	A: 6 B: 31 C: 69 D: 46
Ground truth	Malignant: 42 Benign: 114 Normal: 274	Malignant: 42 Benign: 30 Normal: 80
Histology of malignant lesions (42 lesions in 42 patients)	Invasive ductal carcinoma: 32 Ductal carcinoma in situ: 3 Mucinous carcinoma: 2 Intraductal papillary carcinoma: 2 Metaplastic carcinoma: 1 Neuroendocrine carcinoma: 1 Apocrine carcinoma: 1	Invasive ductal carcinoma: 32 Ductal carcinoma in situ: 3 Mucinous carcinoma: 2 Intraductal papillary carcinoma: 2 Metaplastic carcinoma: 1 Neuroendocrine carcinoma: 1 Apocrine carcinoma: 1
Histology of benign lesions (Full Cohort: 152 lesions in 114 patients, 38 patients had two benign lesions; Observer Study Cohort: 35 lesions in 30 patients, 5 patients had two benign lesions)	Fibroadenoma: 79 Cyst: 1 Intraductal papillary: 11 Radial scar: 1 Atypical Ductal Hyperplasia: 6 Lipoma: 1 Phyllodes tumor: 2 Hamartoma: 1 Sclerosing adenosis: 2 Benign hyperplasia: 48	Fibroadenoma: 14 Intraductal papillary: 3 Atypical Ductal Hyperplasia: 2 Lipoma: 1 Phyllodes tumor: 2 Hamartoma: 1 Benign hyperplasia: 12

When available, median and range (within parentheses) are given

*BI-RADS* Breast imaging reporting and data system, *DM* Digital mammography

The DM exams were acquired with GE Senographe Essential mammography systems and consisted of two image views per breast (cranio-caudal and medio-lateral oblique). The ABUS scan data were obtained by trained technicians or nurses using a standardized scanning protocol, using a GE Invenia ABUS system. Patients were positioned supine on an examination bed with the ipsilateral arm above the head. Each breast was scanned by an automated 6–14 MHz linear transducer (covering an area of 15.4 × 17.0 × 5.0 cm) in three standard image views: lateral, anteroposterior and medial.

### Single-modality AI systems

Each case was automatically processed by two commercially available AI systems developed for fully automated breast cancer detection, one for DM (Transpara® 1.7.0, ScreenPoint Medical BV, Nijmegen, The Netherlands), further referred to as AI-DM and one for automated 3D breast ultrasound ABUS (QVCAD 3.4, Qview Medical Inc., Los Altos, California, USA), referred to as AI-ABUS. Both systems (AI-DM and AI-ABUS) use machine learning algorithms to detect areas suspicious of breast cancer in the images, and research studies have demonstrated that these systems can improve radiologists’ reading performance when concurrently used for support [13, 16].

The systems provide a continuous score per exam representing the risk that breast cancer is present in any of the images/volumes of the examination.

### Multi-modality AI system

The scores from both AI-DM and AI-ABUS were combined at a case-level to create a multi-modal AI system, referred to as AI-Multimodal. No region information was taken into account. The scores of the single modality AI systems were first normalized to 0 and 1, and then different weights (ranging from 0.01 to 0.99, the sum of 1) were applied on the two scores, resulting in a continuous suspicion score between 0 and 1 for AI-Multimodal for every case.

### Benchmarking observer study

For benchmarking, a subcohort of the collected full dataset was selected for an observer study aiming to measure radiologists’ performance evaluating DM and ABUS in a population of women with predominantly dense breasts. The subcohort used for this observer study consisted of all 42 malignant cases and a total of 30 benign and 80 normal, randomly chosen cases (in order to include approximately 2 normal exams for each malignant case and a sample of benign cases that represents approximately 20% of the total). More details can be found in Table 1.

Two radiologists (7 and 10 years of experience reading DM) independently read DM-only exams and two other radiologists (4 and 6 years of experience reading ABUS) independently read ABUS-only exams using Breast Imaging Reporting and Data System (BI-RADS®) scores (category 1 = negative; category 2 = benign; category 3 = probably benign; category 4 including 4a, 4b, 4c = suspicious; category 5 = highly suggestive of malignancy). The radiologists had no time constraint, used certified medical workstations, and were blinded to the patient’s identity, results of other modalities and medical background.

As a result of the observer study, different operating points of radiologists (and their combination) were computed for benchmarking of AI-DM, AI-ABUS, and their combination AI-Multimodal against:

- Single human reading of DM or ABUS
- Average of two readers reading the same modality (DM or ABUS). A case was presumed to be recalled if either reader recalled the case.
- Average of two readers reading complementary modalities (one DM, one ABUS). A case was presumed to be recalled if either reader recalled the case.
- Panel of four readers (two reading DM, two reading ABUS). Majority voting was used to simulate the BIRADS score of the case.

### Statistical analysis

Receiver operating characteristic (ROC) curves and the area under the curve (AUC) values including 95% confidence intervals (CI) were computed and compared for AI-DM, AI-ABUS, and AI-Multimodality using the DeLong method [21] in the full data cohort.

In all analyses, only cases with biopsy-proven malignant lesions were considered positive. *p*-values < 0.05 were considered significant. The maximum Youden’s index and its associated sensitivity and specificity are also reported for each AI system.

Detection performance of human readers (and their simulated combinations as described in the previous section) in the subcohort of the reader study was measured in terms of sensitivity and specificity. 95% CI were computed using bootstrapping.

## Results

### AI-detection performance on the full cohort

**Fig. 1:**
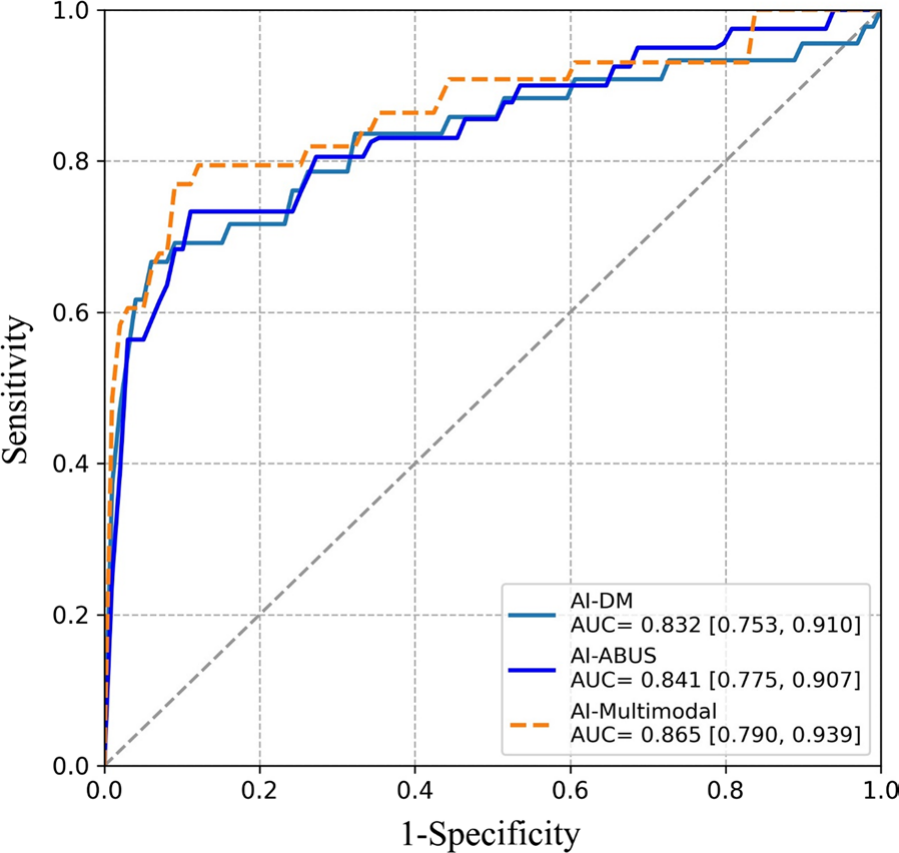
Receiver operating characteristic curve comparison (and their area under the curve, AUC) among AI-DM, AI-ABUS and AI-Multimodal in full cohort

In the full cohort, the optimal AUC of the AI-Multimodal was 0.865 (95% CI: 0.790, 0.939) using weights of AI-DM and AI-ABUS of 0.25 and 0.75, respectively. This was higher than the AUC of the AI-DM alone (0.832, 95% CI: 0.753, 0.910) (*p* = 0.026) and AI-ABUS alone (0.841, 95% CI: 0.775, 0.907) (*p* = 0.041). The ROC curves are displayed in Fig. 1. The maximum Youden’s index for AI-DM was 0.650 (sensitivity and specificity of 76.1% and 88.9%), for AI-ABUS was 0.680 (sensitivity and specificity of 80.8% and 87.2%), and for AI-Multimodal was 0.707 (sensitivity and specificity of 79.4% and 91.2%).

### AI benchmarking against radiologists performance

**Fig. 2:**
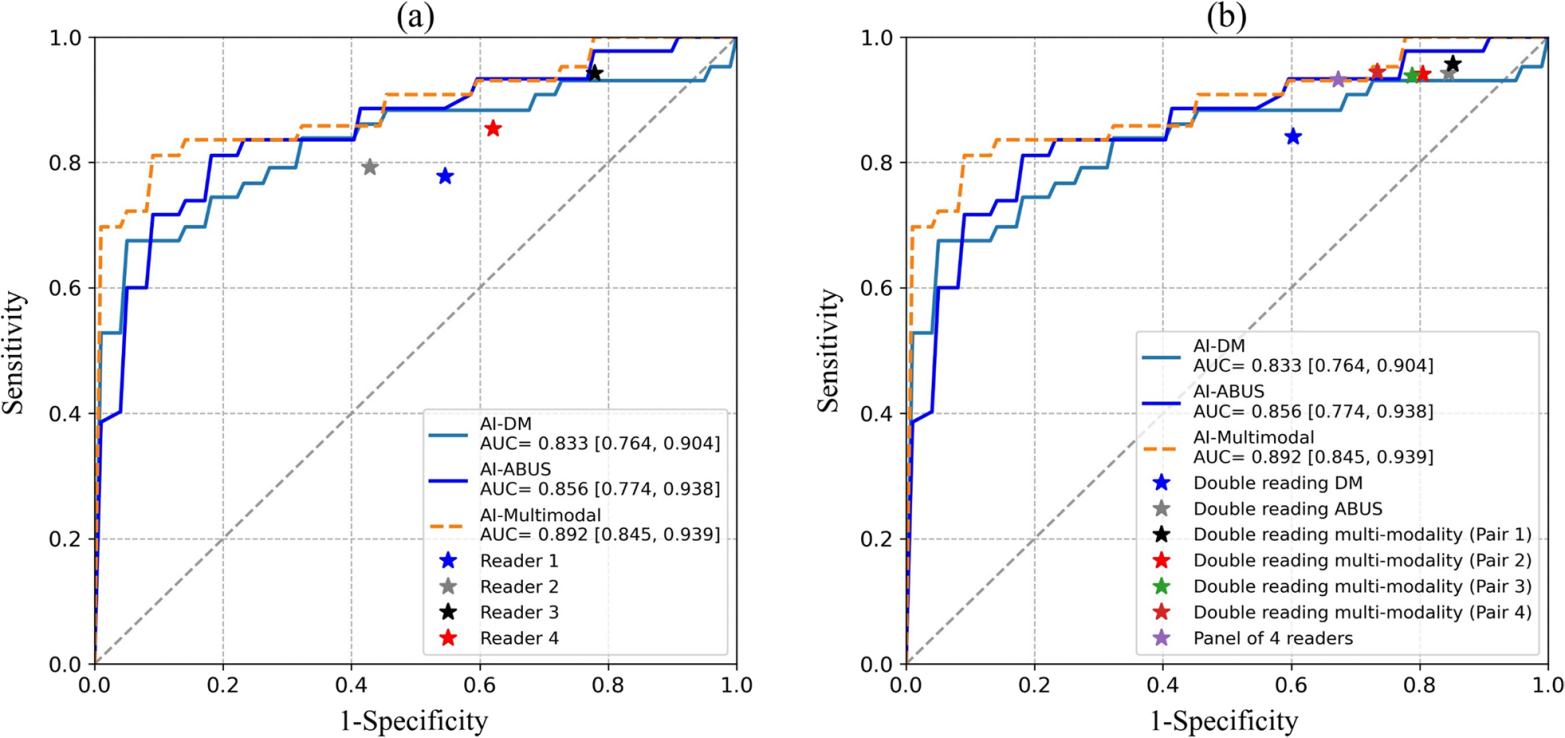
Operating point of radiologists and receiver operating characteristic curve (ROC) comparison (and their area under the curve, AUC) in observer study cohort. **a** the ROCs of AI-DM, AI-ABUS and AI-Multimodal, and the operating point of original readers; **b** the ROCs of AI-DM, AI-ABUS and AI-Multimodal, and the operating point of simulated pairs and the panel of 4 readers

The ROC curves of AI-DM, AI-ABUS, and AI-Multimodality were similar in the subcohort and in the full cohort (Fig. 2). When the ROC curve of AI-Multimodal is overlayed with the operating points of radiologists, it is observed that AI-Multimodal achieves superior or equal sensitivity as single human readers at the same specificity operating points.

**Fig. 3:**
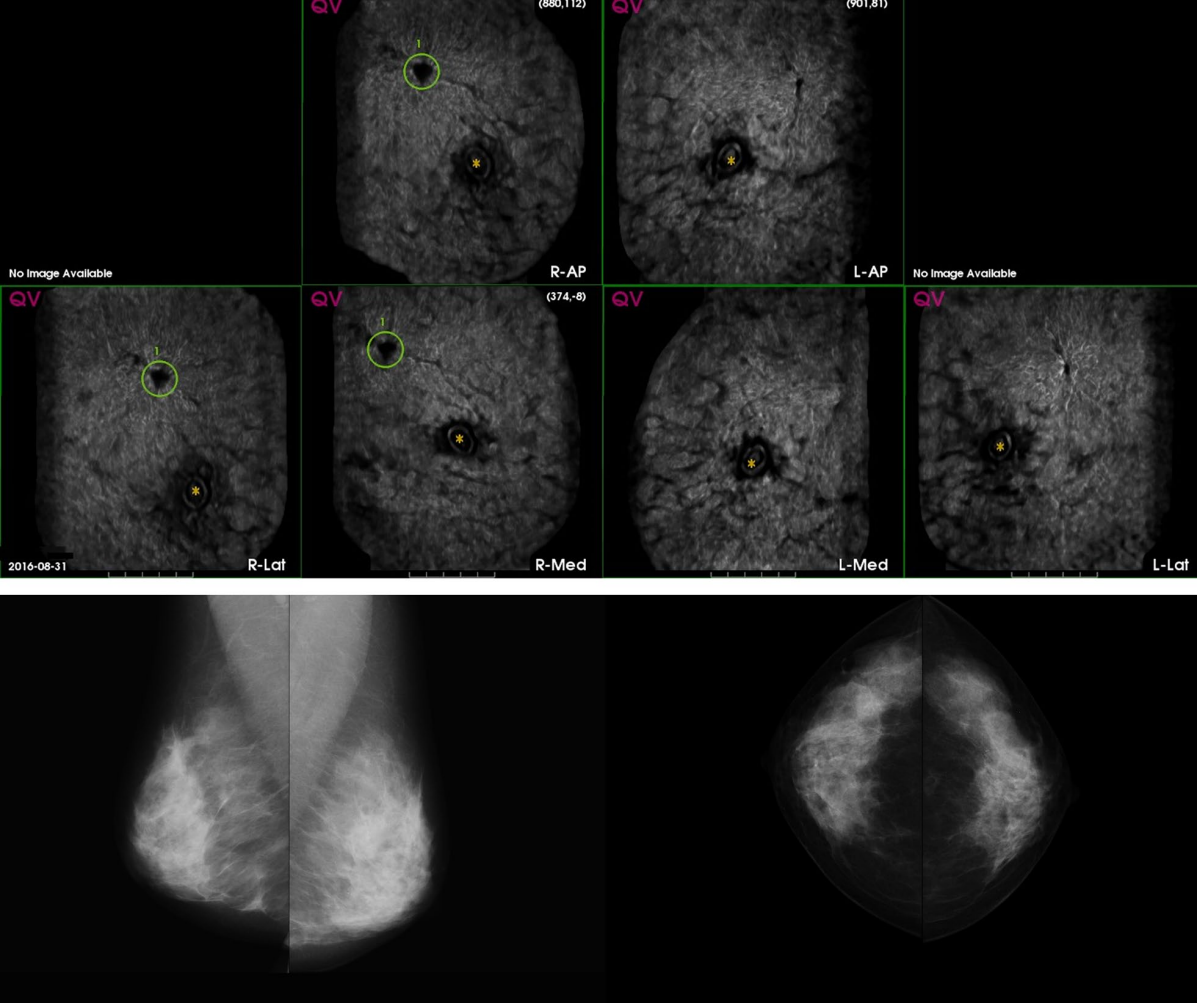
ABUS (top) and DM (bottom) examination of a woman 44 years old with a diagnosed invasive ductal carcinoma 2 cm grade 3, right breast. AI-ABUS detected the cancer lesion with a high score (99.9, scale 1–100), which was not detected by AI-DM (score of 37.8, scale 1–100) or the radiologists (BI-RADS 2 and BI-RADS 3, respectively)

**Fig. 4:**
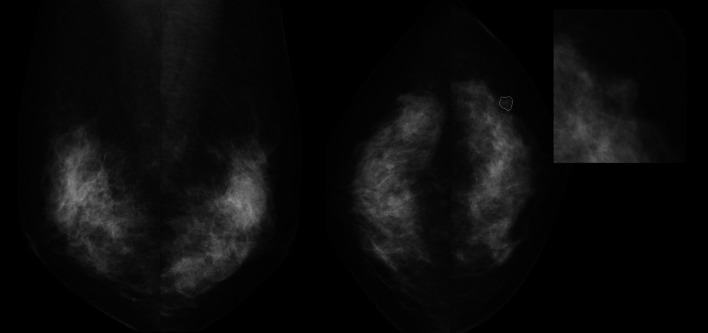
DM examination of a woman 50 years old without any diagnosed pathology. AI-DM yielded a false positive assessment since it assigned a high score to the case (92.6, scale 1–100) based on the delineated suspicious density, both radiologists assigned BI-RADS 3 to the case while AI-ABUS assigned a lower score (76.1, scale 1–100) showing the potential of AI-ABUS to also reduce the false positives of AI-DM

The sensitivity and specificity of AI systems and of the real and simulated radiologists combinations are presented in Table 2. The applied double reading strategies improve performance over single reading. The use of ABUS over a strategy with DM alone is clearly beneficial. The panel of four readers achieved a sensitivity of 93.2% and specificity of 32.7%. At the same specificity, AI-Multimodal obtained equal sensitivity. At the Youden’s index in this subcohort, AI-Multimodal yielded a sensitivity of 81.1% and specificity of 95.5%. Case examples of the study are shown in Figs. 3 and 4.

**Table 2:** The sensitivity and specificity values

	Sensitivity (%)	Specificity (%)
AI-DM (Maximum Youden’s index)	77.2 [60.1, 94.4]	89.1 [79.1, 99.0]
AI-ABUS (Maximum Youden’s index)	86.1 [78.9, 93.2]	82.7 [71.6, 93.8]
AI-Multimodal (Maximum Youden’s index)	81.1 [73.4, 88.8]	95.5 [91.9, 99.0]
Single reader DM (Reader 1)	77.8 [70.0, 85.6]	45.4 [37.4, 53.5]
Single reader DM (Reader 2)	79.2 [75.1, 83.2]	57.1 [53.1, 61.2]
Single reader ABUS (Reader 3)	94.2 [93.1, 95.3]	22.1 [19.1, 25.2]
Single reader ABUS (Reader 4)	85.4 [81.1, 89.6]	37.9 [35.4, 40.5]
Double reading DM	84.1 [79.3, 88.9]	39.7 [33.0, 46.5]
Double reading ABUS	94.2 [93.1, 95.3]	15.5 [12.6, 18.3]
Double reading multi-modality (Pair 1)	95.7 [93.7, 97.7]	14.8 [11.5, 18.1]
Double reading multi-modality (Pair 2)	94.1 [93.3, 95.0]	19.5 [13.6, 25.4]
Double reading multi-modality (Pair 3)	93.9 [93.1, 94.7]	21.2 [17.7, 24.8]
Double reading multi-modality (Pair 4)	94.4 [93.5, 95.3]	26.6 [20.9, 32.3]
Panel of 4 readers	93.2 [89.2, 97.3]	32.7 [23.9, 41.6]

Sensitivity and Specificity (including 95% confidence intervals) values of radiologists and artificial intelligence (AI) systems in digital mammography (DM), automated 3D breast ultrasound (ABUS), and the multi-modality simulation for the observer study cohort

## Discussion

Our results show the potential performance benefits of combining artificial intelligence algorithms from digital mammograms (DM) and automated 3D breast ultrasound (ABUS) in a population with mostly dense breasts.

Without the involvement of radiologists, incorporating AI results from ABUS into an existing AI system for mammograms boosts the diagnostic performance and outperforms the performance of single readers on mammography. This appears to be independent from the presence of biopsied benign lesions in the dataset. This could be used to automatically increase the detection performance in screening, without an additional burden in reading time per exam, albeit the scans obviously still need to be obtained. Future assessments in a real screening environment are needed to determine the benefits and problems of such integration, since an ABUS acquisition adds costs to the screening workflow.

When comparing the AI performance to radiologists reading only DM or ABUS, we observed that the AI systems analyzing also only DM or ABUS achieves a similar performance. The AI system for mammography used in this study was already shown to perform as good as the average of radiologists in Europe and the US [17]. This study shows a similar result for the evaluated population of Chinese women and radiologists. ABUS has a somewhat higher sensitivity than mammography in this population, at a lower specificity. We observed that the performance of AI-ABUS is similar to that of AI-DM and to the radiologists reading only ABUS. The proposed weighted combination of AI-DM and AI-ABUS improves the AI performance and appears to be as good as 4 radiologists in consensus (2 reading mammography and 2 ABUS). This may enhance the complementary value of AI in reading multi-modal screening examinations, and may provide a possibility to use AI as a stand-alone reader in radiologists’ scarce regions with populations with dense breasts that could benefit from ABUS.

Compared to daily routine and screening situations, the reader study was enriched with cancer cases and benign cases. Therefore, human performance may have been affected by a “study effect” that reflects the reading of such enriched datasets [22]. It should also be noted that the cases only included soft tissue lesions only, not calcifications, and that correct lesion localization was not taken into account, albeit previous work showed good localization accuracy of the AI systems. Statistically, a limitation of the study is that scores were combined by averaging (following the work by Wu et al. [23]), while the actual benefit of combining radiologists and AI depends on the interaction functionalities. It may be expected that when humans interact with AI, the effect is more complex than a simple averaging of independent computed scores. However, our study suggests that there is potential to complement human thinking with AI computations to improve the performance of radiologists. In some settings both elements might, however, be used independently (AI in the background plus radiologists unaided), a situation for which our results are more representative. For example, a potential scenario could be to have AI on ABUS images working in the background to alert readers (initially only assessing the mammograms) about likely suspicious findings or likely normal cases. This scenario might be particularly relevant in a setting where different readers assess mammography and ABUS, as the AI system might indicate a strong need to assess the ABUS scan, whereas it could also indicate likely normal cases in which reading of the ABUS examination could be skipped, thus saving time and resources. The operating points at which such decisions can be made are a topic of future research.

The current AI systems were shown to be as good as experienced radiologists in a top-ranked (Grade-A Tertiary) Hospital which only accounts for 3.0% of Chinese hospitals. However, the mortality rate from breast cancer in areas where experienced radiologists are lacking is much higher, partially due to delayed diagnosis and partially due to treatment differences. In China, radiologists reading mammograms and radiologists reading ultrasound images belong to two separate departments [24]. They usually lack experience in reading the other modality images. Therefore, multi-modal AI can be of help for combining information from the two modalities. For such experienced radiologists-lacking regions, direct implementation of a multi-modal AI systems may benefit patient care. In other words, the multi-modal AI system could be directly used as an independent stand-alone reader to improve the diagnosis for cancers, with great potential in rural areas where radiologists are in extreme scarcity and there are needs to save human resources of medical centers, provided that it is possible to train technicians to utilize the mammography and ultrasound machines. The main message from our study is therefore that AI-DM/AI-ABUS can perform as good as experienced radiologists in an Asian population and that by combining AI systems, breast cancer diagnostics can be further improved.

There are some limitations in our study. First, in the observer study, two readers were only for DM, the other two were only for ABUS, and readers did not read both modalities at the same time because of their respective specialties. Second, this is only a single-center study and future studies shall involve external data to validate the generalizability of our multi-modal AI system.

## Conclusion

In a population of women with predominantly dense breasts, combining AI results from DM and ABUS improves the performance of a single-modality AI (which in the case of DM/ABUS is equivalent to a radiologist), while the addition of automated multi-modality AI readings might boost the performance of radiologists’ alone. The multi-modal AI system for detecting breast cancer in women with dense breasts may be used an aid tool for breast screening in radiologist-scarce regions. Although promising, the best way of deploying the AI systems and their combination in a multi-modal screening setting remains to be further investigated. Clinically relevant end-points, such as the number of avoided benign biopsies or the improvement in detection sensitivity should be further evaluated.

## Data Availability

The datasets used and/or analyzed during the current study are available from the corresponding author on reasonable request.

## Abbreviations

ABUS: Automated 3D breast ultrasound
AI: Artificial intelligence
AUC: Area under the receiver operating characteristic curve
BI-RADS: Breast imaging reporting and data system
CAD: Computer-aided detection
DM: Digital mammography
FDA: Food and drug administration
ROC: Receiver operating characteristic
